# Antipsychotic medications and cognitive functioning in bipolar disorder: moderating effects of COMT Val^108/158^ Met genotype

**DOI:** 10.1186/1471-244X-13-63

**Published:** 2013-02-19

**Authors:** Baer Arts, Claudia JP Simons, Marjan Drukker, Jim van Os

**Affiliations:** 1 Department of Psychiatry and Psychology, School for Mental Health and Neuroscience, European Graduate School of Neuroscience (EURON), South Limburg Mental Health Research and Teaching Network (SEARCH), Maastricht University Medical Centre, P.O. Box 616 (DRT 12), Maastricht, MD, 6200, The Netherlands; 2 GGZE, Institute for Mental Health Care Eindhoven en de Kempen, P.O. Box 909, Eindhoven, AX, 5600, The Netherlands; 3 King’s College London, King’s Health Partners, Department of Psychosis Studies, Institute of Psychiatry, London, United Kingdom

**Keywords:** Bipolar disorder, Cognition, Antipsychotics, COMT

## Abstract

**Background:**

There is a negative association between the use of antipsychotics and cognitive functioning in bipolar patients, which may be mediated by altered dopamine signaling in selected brain areas, and moderation thereof by genetic sequence variation such as COMT Val^108/158^Met. The interaction between antipsychotic drug use and the COMT Val^108/158^Met genotype on two-year cognitive functioning in bipolar patients was examined.

**Methods:**

Interaction between the COMT Val^108/158^Met and antipsychotics on a composite cognitive measure was examined in 51 bipolar patients who were assessed 12 times at two-monthly intervals over a period of two years (379 observations).

**Results:**

There was a significant negative effect of the interaction between antipsychotic medications and Val allele load on the composite cognitive measure in bipolar patients (p < 0.001).

**Conclusions:**

The negative effects of antipsychotics on cognitive functioning in bipolar disorder may be moderated by the COMT Val ^108/158^ Met genotype, with a negative effect of Val allele load. If replicated, the results may be indicative of pharmacogenetic interactions in bipolar disorder.

## Background

Meta-analyses of neuropsychological functioning in euthymic bipolar patients suggest that generalized, rather than specific, cognitive impairments may exist, characterized by substantial heterogeneity that is not fully explained by demographic, illness and medication variables [1-4]. Nevertheless, reviews point to a possible role for antipsychotics, indicating that antipsychotics may have detrimental impact on cognitive functioning in bipolar patients [5]. For example, Jamrozinski and colleagues (2008) reported no differences between euthymic bipolar patients not exposed to antipsychotic treatment and healthy controls on any neuropsychological measure, whereas a significant underperformance was apparent in the bipolar group treated with antipsychotics [6]. A more recent study showed dose-independent deficits in several cognitive tasks in euthymic bipolar patients treated with quetiapine, olanzapine or risperidone, with worse performance in patients on second generation antipsychotics compared to untreated euthymic patients [7]. A recent 2-year naturalistic study on cognitive functioning in bipolar patients showed significant variation of cognitive functioning over time, largely independent of clinical factors, with the exception of antipsychotic drug use impacting negatively on tasks indexing speed of information processing [8]. The suggested negative effects of antipsychotics on cognition in bipolar patients contrast with the apparently positive, cognition-enhancing effects of these drugs in the treatment of schizophrenia and schizoaffective disorder [9-12]. However, cognition-enhancing effects in schizophrenia may at least in part be attributable to practice effects [13,14]. Cognitive effects of antipsychotics may be mediated by alterations in dopamine signaling in selected brain areas. It may be hypothesized that in altered and/or hyperdopaminergic states, which may underlie schizophrenia symptoms [15], antipsychotic drugs improve cognition, whereas in bipolar patients without hyperdopaminergia, antipsychotic treatment may induce suboptimal cognitive functioning [6].

Antipsychotic effects impacting on dopamine signaling may be modulated by genetic sequence variation. The COMT (catechol-O-methyltransferase) gene Val^108/158^Met polymorphism modulates dopaminergic function in frontostriatal circuitry, and may impact information processing efficiency, due to its critical role in the enzymatic degradation of dopamine. The Val/Val genotype is associated with greater activity of the enzyme and hence with lower concentrations of dopamine in the prefrontal cortex [16,17]. Furthermore, in patients with schizophrenia, an interaction of the COMT Val^108/158^Met genotype and antipsychotic treatment on cognitive functioning has been reported, Met allele load predicting better cognitive performance [18-21], especially in tasks requiring effortful cognitive control [22].

The aim of the present study was to examine whether COMT Val^108/158^Met genotype modulates the effect of antipsychotics on a composite cognitive measure, indexing effortful cognitive control, in a sample of bipolar patients, hypothesizing a detrimental effect of Val allele load.

## Methods

### Subjects

Individuals were participants in the BIPOLCOG (BIPOLar and COGnition) study [23], a study on cognitive functioning in bipolar disorder (BD) in which three groups were investigated: (i) patients with bipolar disorder, (ii) healthy first-degree relatives of patients with bipolar disorder, and (iii) healthy control participants. All subjects were white, between the ages of 18 and 60 years, fluent in Dutch, had an IQ > 70 and were without a history of neurological disorders such as epilepsy and concussion with loss of consciousness. For the purpose of the current report, only the bipolar patient group was studied, with the healthy control group as reference.

A representative cohort of successively attending patients with a diagnosis of bipolar spectrum disorder according to DSM-IV [24] were recruited through the in-patient and out-patient mental health facilities in the geographically defined catchment area of South Limburg. In addition, patients were recruited through the local association of bipolar patients and their families, in order to also include patients not currently in contact with services. The computer program OPCRIT was used to confirm DSM-IV diagnosis on the basis of current and lifetime recorded symptomatology listed in the Operational Criteria Checklist for Psychotic Illness, scored by the clinical researcher on the basis of all interview and historical case note data (OCCPI) [25].

Control subjects were recruited from the general population using a random mailing sampling procedure and were clinically and diagnostically interviewed with The Comprehensive Assessment of Symptoms and History (CASH) [26] and OPCRIT criteria to exclude those with a past or current diagnosis of BD or psychotic disorder. Healthy controls were additionally interviewed with the Family Interview for Genetic Studies (FIGS) [27] in order to confirm the absence of a family history of psychotic or bipolar disorder.

The initial sample consisted of 81 patients and 61 healthy control subjects. Three patients were excluded because data on diagnosis were missing. Neuropsychological testing data were missing for two patients. The last filter concerned incomplete or missing genetic data, leaving a final risk set for analysis of 51 patients and 50 healthy controls.

### Procedure

As cognitive alterations in bipolar disorder largely develop after onset of illness [28], longitudinal assessment is necessary to adequately capture the phenotype. Thus, patients were examined at 2-monthly intervals over a period of 2 years, yielding a maximum of 12 assessments. At all time points, neuropsychological testing and psychiatric interviewing took place and questionnaires were completed (regarding social functioning, medication, use of drugs etc.). Genetic material was collected at the first visit. During the baseline interview, basic demographic information was collected as was information on illness characteristics.

The study was performed in conformity with the Declaration of Helsinki, and approved by the Ethics Committee of the Maastricht University and Academic Hospital. All subjects gave written informed consent prior to participation.

Neuropsychological tests and psychiatric interviews were conducted by trained psychologists, each interview occasion taking approximately 2 hours to complete.

Healthy controls, in whom cognition is more stable than in patients, were tested twice at two monthly intervals. Data on healthy controls were used as reference to calculate standardized z-scores.

### Neurocognitive assessment

Neurocognitive tests were administered by computer, using E-prime for Windows on a 15-inch monitor Toshiba Tecra laptop. The test battery included tasks measuring various neurocognitive domains, guided by previous evidence of impaired performance in these domains in bipolar patients [1,2]. Three subtests were selected *a priori* from the original test battery, described elsewhere [8], representing tasks with high cognitive load that previous work suggests are most sensitive to moderation by sequence variation in COMT [29-31].

Overall intellectual functioning was estimated at baseline using three Groningen Intelligence Test (GIT) subtests (Mental Rotation, Word Analogies and Mental Arithmetic) [32], yielding results that are comparable to those of the Wechsler Adult Intelligence Scale III [33].

Verbal learning and memory was assessed with the standardized Dutch version of the visually-presented Verbal Learning Test [34,35]. In three consecutive trials, 15 monosyllabic non-related words had to be memorized and reproduced. Delayed recall was measured after a 20-minute delay. Parallel versions of this test were used, in order to avoid test-retest-effects.

The Flanker CPT (Cogtest plc, London) [36] is a measure of selective visual control of attention. Subjects are instructed to respond by pressing the right or left mouse button depending on whether the middle element in a display of five lines has an arrowhead pointing to the right or left. There are three trial types: (i) neutral trials in which the flankers are just horizontal lines without arrowheads, (ii) congruent trials in which all flankers have an arrowhead pointing in the same direction as the target, and (iii) incongruent trials, in which flankers are pointing in the opposite direction from the target. The incongruent condition involves more cognitive effort, because the flankers are associated with a response that needs to be suppressed (measure of response inhibition). One-half of the trials was presented with the stimuli above the fixation cross and the other half were presented below fixation, in order to prevent the subjects from keeping their gaze fixed in one position. The test consisted of 144 trials of neutral, congruent and incongruent flankers, which were presented randomly. Outcome measure was the mean reaction time for correct responses (RT) in the incongruent condition.

Finally, Digit Span Backward of the Wechsler Intelligence Scale III [37] was used as measure of working memory.

All 3 cognitive measures were standardised, higher scores reflecting better performance. In order to calculate a measure of global cognitive functioning, raw test scores were converted into standardized z-scores against the means and standard deviations of the healthy control group. The final composite measure of neurocognition was based on the means of the three domain scores, representing effortful information processing (verbal memory, selective attention/response inhibition and working memory).

### Genotyping

Buccal swab samples were obtained followed by SNP analysis. For the current analysis, the COMTVal^108/158^Met rs4680 was selected *a priori* because this is the only SNP that is consistently associated with antipsychotics in pharmacogenetic models [18,19], making it the only credible candidate. Although more SNPs had been determined (n = 184), these formed part of a standard set for the study of gene-environment interactions (GxE), based on published findings up to April 2009 (for the rational underlying this selection process and an overview of selected SNPs see [38]). The 184 SNPs had been chosen *a priori* (i.e. were not selected from a larger set of genome-wide markers) and selectively determined by Sequenom (Hamburg, Germany) using the Sequenom Mass ARRAY iPLEX platform at the facilities of the manufacturer. In accordance with *a priori* quality control criteria of the GROUP study, SNPs with more than 10% genotyping errors were excluded, as were SNPs in marked Hardy–Weinberg disequilibrium (p <0.001).

### Statistical analysis

Regression analyses were carried out using the statistical software program STATA (version 11.2) [39]. In the bipolar patient group, the moderating effect of Val allele load on the cognitive effects of antipsychotic medications was analysed on the 3 separate cognitive tasks, as well as on the composite cognitive measure. The Bonferroni correction for multiple testing was applied, yielding a corrected p-value for significance of p < 0.00625 (0.05/8).

Data were hierarchical with multiple observations (interview occasion or time; level 1) clustered within subjects (level 2). Data, including the demographic and cognitive data, were analysed using the STATA XTREG multilevel regression routine. The analyses of the interaction between the COMT Val allele load and antipsychotics on cognitive functioning were *a priori* adjusted for the confounding effects of demographic characteristics (age, sex, education), symptoms (BPRS, HDRS, YMRS), and time as fixed factors.

Dummies were constructed for Val allele load with value 0 for the Met/Met genotype and value 1 for the combination of the Val/Met and Val/Val condition. The Val/Met and Val/Val conditions were combined because of the small number of observations in the Val/Val condition. Finally, dummies were constructed for the medication variable with value 1 for using antipsychotics and value 0 for not using this type of medication. Stratified effects were calculated from the model containing the interaction, using the STATA MARGINS command.

### Power analysis

Power of the analysis was calculated by simulation from an example on the Stata website (http://www.stata.com/support/faqs/stat/power.html). Because these simulations cannot be performed in multilevel data, the unilevel equivalent of the n of the multilevel data set was calculated using the following formulaes [40].

(1) $$\mathrm{MF}=1+\left(9–1\right)*0.6=5.8$$

(2) uen=51*9/5.8=79

In which 9 is the number of assessments per person, 51 is the number of persons and 0.6 is the intra class correlation. The power of our sample size of n = 79, alpha = 0.00625 and a large effect size (0.8 sd) was 0.10.

## Results

### Subjects

Demographic data, symptom scores and neurocognitive test results are presented in Table 1. There were no significant differences between the two groups (Met/Met versusVal/Met plus Val/Val) after Bonferroni correction (p < 0.00625). However, the combined Val/Met plus Val/Val group performed better on the working memory task than the Met/Met group (beta: 1.15; p = 0.033, i.e. not Bonferrone corrected). Thirteen of the 51 patients used antipsychotic medications during the two-year period of our study; these patients contributed 38 observations. Allele frequencies were 56% for the Met allele and 45% for the Val allele, respectively.

**Table 1:** Demographics, psychopathology and neurocognitive testresults of COMT Val/Met rs4680 in bipolar patients

	**Met/Met (N = 12; 92 obs.)**		**Val/Met + Val/Val (N = 39; 355 obs.)**	
	**Mean**	**SD**	**Mean**	**SD**
Gender M/F	7/5		20/19	
Age	45.5	8.7	46.5	6.8
Education	4.6	2.4	5.5	2.2
BPRS^1^	1.3	0.2	1.3	0.2
HDRS^2^	2.9	3.6	3.3	3.9
YMRS^3^	2.1	3.2	1.1	2.3
GIT-IQ	101.7	9.7	110.5	13.3
Verbal memory	7.2	2.4	8.2	3.7
Selective attention	729.5	77.4	687.6	81.9
Working memory	5.7	1.6	6.9	2.1
Antipsychotic use	5		8	

^**1**^ Brief Psychiatric Rating Scale.

^**2**^ Hamilton Depression Rating Scale.

^**3**^ Young Mania Rating Scale.

### Genotype

Twelve patients were homozygous for the Met allele, 32 patients were heterozygous and 7 patients were homozygous for the Val allele. Genotypes in the bipolar group were in Hardy-Weinberg equilibrium (P > 0.09).

### Cognitive data

The interaction between COMTVal^108/158^Met Val allele load and antipsychotics on two-year cognitive functioning in bipolar patients is presented in Table 2. As evidenced in this table, there exists a detrimental effect of Val allele load on the cognitive effects of antipsychotic medications. The composite cognitive measure survived Bonferroni correction.

**Table 2:** Interaction between antipsychotic medications and COMT Val/Met polymorphism (rs4680) on cognitive functioning in bipolar patients (Met/Met as reference; psychopathology and time as fixed factors)

**Cognitive**	**Variable**	**Verbal**	**Memory**	**Selective**	**Attention**	**Working**	**Memory**	**Composite**	**Measure**
N obs.		419		384		416		379	
		**beta**	**p-value**	**beta**	**p-value**	**beta**	**p-value**	**beta**	**p-value**
COMT		0.24	0.511	0.63	0.053	0.5	0.051	0.42	0.036
Antipsych.		−0.03	0.932	0.64	0.087	0.05	0.858	0.19	0.367
MainInteract.		−0.79	0.091	−1.1	0.024	−0.35	0.340	−0.79	0.004
Stratified	COMT = 0			0.64	0.087			0.19	0.367
interaction	COMT = 1			−0.47	0.141			−0.59	0.001
BPRS		−0.05	0.492	−0.02	0.835	−0.05	0.396	−0.04	0.385
HDRS		0.005	0.948	0.05	0.551	0.06	0.292	0.02	0.602
YMRS		0.002	0.972	0.12	0.029	0.07	0.061	0.06	0.063
Time		0.06	0.000	0.13	0.000	0.09	0.000	0.09	0.000

## Discussion

### Summary of findings

Patients with bipolar disorder displayed a negative modulating effect of COMT Val^108/158^Met Val allele load on the effects of antipsychotics on two-year cognitive functioning. Given the small number of patients, this finding must be considered preliminary. Replication of this underpowered, hypothesis-generating study, would require a number of 193 patients in order to obtain a power of 0.8 (given the small number of patients using antipsychotics). Alternatively, increasing the number of patients using antipsychotics would increase power too. Nevertheless, the results, if replicated, suggest a gene-by-environment interaction between antipsychotic medications and COMT Val^108/158^Met rs 4680. Patients with a more severe course of illness may be more likely to receive antipsychotic medications; however, *post*-*hoc* analyses revealed no significant effects of number of episodes and/or psychotic symptoms in the past on the interaction between antipsychotics and Val allele load. Furthermore, *post*-*hoc* analyses yielded no significant interactive effects of Val allele load on tasks requiring less cognitive effort, such as sustained attention and motor speed.

### Interaction between antipsychotics and COMT Val^108/158^Met on cognitive functioning

Our finding of a negative effect on cognition of antipsychotics in Val allele carriers is in line with the sparse literature in patients with schizophrenia. Rebollo-Mesa et al. (2011), for instance, report a negative effect on cognition in Val/Val homozygotes using antipsychotics in contrast with a reversed association in Met/Met carriers [21]. Furthermore, a positive effect of antipsychotics on cognitive functioning in Met allele carriers with schizophrenia is described in the literature, with no evidence of improvement of cognition in Val allele carriers using antipsychotics, thus indicating interactive effects [18-20]. These findings support the hypothesis of Jamrozinski et al. (2009) of differential effects of antipsychotics on cognitive functioning depending on basal dopamine levels, antipsychotics lowering dopamine functioning and inducing suboptimal cognitive status in bipolar patients with already lower basal dopamine levels, as is the case in Val allele carriers [6].

### COMTVal^108/158^Met rs4680 and cognitive functioning

In general, the COMT Val^108/158^Met polymorphism appears to have little if any direct association with cognitive functioning [41], with mixed results in patients with schizophrenia [42,43] and bipolar disorder [29,31], as well as in healthy controls [44,45]. Krabbendam et al. (2006) suggest indirect (positive) effects of Met allele loading on cognitive functioning, independent of schizophrenia liability, through gene-gene interactions or the influence of a functional polymorphism near COMT Val^108/158^Met [46]. The latter is illustrated by the study of Burdick et al. (2007), finding no association between COMTrs4680 and cognitive functioning, but an association between cognitive functioning and COMTrs165599 in bipolar patients and healthy controls [47]. Furthermore, Diaz-Asper and colleagues (2008) report a negative effect of the Val allele on cognitive functioning, irrespective of diagnosis, and significant effects on cognition of other COMT haplotypes, for instance COMTrs737865 [30]. Finally, the literature supports the possible role of gene-gene interactions on cognitive functioning due to epistatis between COMT and several other gene polymorphisms, impacting dopamine signalling [48-50], GABAergic functioning [51,52] and glutamatergic systems [53,54], amongst others.

In conclusion, COMT most likely has indirect effects on cognitive functioning by modulating dopaminergic neurotransmission, influencing attentional network efficiency [55], amongst others, with a possible advantage of the Met allele on effortful cognitive control or conflict processing, not supporting a simple stability/flexibility model of dopamine COMT Val/Met effects, according to Rosa et al. [22].

### COMTrs4680 and dopamine function

The dual state, or tonic-phasic dopamine theory hypothesizes that the COMT Met allele results in increased levels of dopamine and a tonic D1-dominated state in the prefrontal cortex, thus increasing signal-to-noise ratio in prefrontal attractor states that may improve performance in some measures, such as working memory, but not others [16,56,57]. The latter is illustrated by the meta-analysis of neuroimaging studies of COMTrs4680 by Mier et al. (2010), reporting a significant association between the COMT genotype and prefrontal activation (effect size: 0.73), with opposing effects for executive cognition paradigms, favouring Met allele carriers, and emotional paradigms, favouring Val allele carriers [58]. These pleiotropic effects of COMT4680 on neural mechanisms underlying cognitive functioning are further illustrated by recent (f-)MRI-studies, showing evidence for COMT-genotype-dependent differences in amygdala responsivity and connectivity [59-61] and prefrontal cortex activation and connectivity patterns, including default network [62-64]. Finally, COMT haplotypes, other than Val/Met, can nonlinearly modulate intelligence-related white matter integrity of the prefrontal lobes by significantly influencing prefrontal dopamine variations, fitting an inverted U-model [65].

### Dopamine and cognitive functioning

The evidence for the inverted U-model of the dopamine actions on cognitive functioning, especially working memory and cognitive control, is reviewed by Cools et al. (2011). These authors conclude that there exists an optimum dopamine level for different cognitive functions, implicating the importance of baseline levels of dopamine, where both too little and too much dopamine may impair performance, depending on a dynamic balance between cognitive stability (prefrontal cortex) and cognitive flexibility (striatum) [66,67]. Prefrontal dopamine D1 receptor activation, in this respect, may improve sustained attention [68], whereas striatal dopamine D2 receptor signalling may be associated with cognitive flexibility [69], with a central role for dopamine in effort-based decision making [70] and the interaction between appetitive motivation and cognition [71], amongst others. The inverted U-shaped curve of dopamine’s action is influenced by (uncontrollable) stress, weakening prefrontal cortex functioning [72], as well as influencing stress-related methylation of the gene, partially compensating the role of the high-activity Val allele in prefrontal cognition [73]. Furthermore, estradiol status and working memory load, which may potentiate dopamine and thus have beneficial effects for Val homozygotes and unfavourable effects for Met homozygotes, may play a role as well [74]. In contrast, Karlsson et al. (2011), found no linear or curvilinear relationships between dopamine D1 receptor binding in dorsolateral prefrontal cortex and performance in any cognitive task, providing support for the notion that D1 receptors in separate brain regions are differentially related to performance in various cognitive tasks [75].

### Methodological considerations

The results should be interpreted in the light of several limitations. First, the small number of patients and observations regarding exposure to antipsychotic medications, makes our results preliminary, needing replication in larger samples. Results therefore can be considered as hypothesis-generating. Second, the effects of possible confounders such as gene-gene interactions, the influence of functional polymorphisms near COMT Val^108/158^Met, epigenetic changes due to stress and/or medication, task demands, and the possible interaction between COMT Val^108/158^Met and herpes simplex virus type 1 infection [29], amongst others, were not adequately controlled for in our study.

The longitudinal character of our study, however, may have been more sensitive to genetic effects, in this case the interaction between COMT Val^108/158^Met Val allele load and antipsychotic medication, given the significant variation of cognitive functioning over time in bipolar patients [8].

## Conclusions

The negative effects of antipsychotic medication on cognitive functioning in patients with bipolar disorder, may be partly moderated by COMT Val^108/158^ Met Val allele load. This finding agrees with an indirect, modulatory role for COMT on (basal) dopamine levels in different brain areas, relevant for (effortful) cognitive functioning, thus influencing the cognitive side-effects of antipsychotics in patients with bipolar disorder.

## Competing interests

Baer Arts none.

Claudia Simons none.

Marjan Drukker none.

Jim van Os is/has been an unrestricted research grant holder with, or has received financial compensation as an independent symposium speaker from Eli Lilly, BMS, Lundbeck, Organon, Janssen-Cilag, GSK, AstraZeneca, Pfizer and Servier, companies that have an interest in the treatment of psychosis.

## Authors’ contributions

All the authors of the article have participated in: 1) making substantial contributions to conception and design, or acquisition of data, or analysis and interpretation of data; 2) writing the drafts of the manuscript or revising them critically for important intellectual content; and 3) giving final approval of the version to be published.

## Pre-publication history

The pre-publication history for this paper can be accessed here:

http://www.biomedcentral.com/1471-244X/13/63/prepub
